# Poliosis as a result of temporary eyelash tinting

**DOI:** 10.1016/j.jdcr.2025.04.029

**Published:** 2025-05-20

**Authors:** Jake Nusynowitz, Seth L. Matarasso

**Affiliations:** a Florida International University Herbert Wertheim College of Medicine, Miami, Florida; b Department of Dermatology, Massachusetts General Hospital, Boston, Massachusetts; c Department of Dermatology, UCSF School of Medicine, San Francisco, California

**Keywords:** cosmetics, eyelash tinting, poliosis

## Introduction

Poliosis is localized hair depigmentation from a loss of melanin in hair follicles that can affect any hair-bearing area. Histology shows reduced or absent melanocytes in affected follicles, while the surrounding epidermis remains intact.^1^ Due to its rarity, the prevalence of poliosis remains unclear. Causes of poliosis include the genetic conditions of Waardenburg syndrome, piebaldism, and tuberous sclerosis. It may also result from autoimmune diseases, including vitiligo, and inflammatory conditions like alopecia areata or trauma,^2^ and it has been linked to medications such as prostaglandin analogs that are used for glaucoma.^1^ Poliosis is often persistent, and in cases caused by inflammation or trauma, partial resolution may occur after treating the underlying condition. However, treatment options remain limited. Repigmentation therapies with phototherapy or topical agents show inconsistent results, leaving many patients to rely on cosmetic tint solutions to camouflage and blend depigmented areas.^3^ This report documents the first case of poliosis occurring after a semipermanent cosmetic eyelash tint procedure.

## Case description

**Fig 1:**
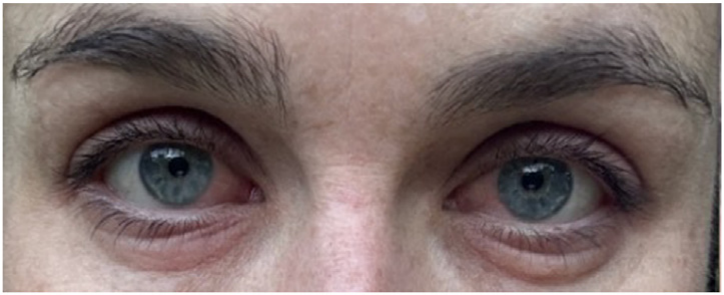
Twenty-four hours postprocedure with periocular edema and erythema in the distribution of the tint.

**Fig 2:**
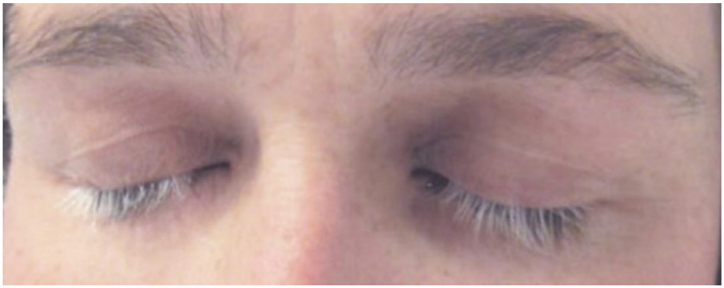
Eight months following the procedure with complete loss of eyelash color.

A 32-year-old woman with no prior medical history and whose sole medication was the long-term use of norethindrone acetate for birth control presented with loss of eyelash pigment following tinting. The patient had received this treatment many times, and neither the protocol nor the product had changed. The periocular area was cleansed with a nonabrasive solution, a petrolatum barrier was applied to the cutaneous eyelid border, and the tint was applied directly from the manufacturer’s package using a brush. The liquid is intended to stay intact for approximately 30 minutes; however, due to a “burning sensation,” it was removed sooner with saline compresses and ocular lavage. Over the next several days, the patient experienced erythema, edema (Fig 1), subsequent desquamation, and conjunctival hyperemia with no visual acuity changes. The patient was not prescribed, nor did she use, any preparations to attenuate the appearance. Approximately 2 weeks after the incident, the patient noted the onset of loss of eyelash color, and at 8 months, roughly 80% of eyelashes were depigmented (Fig 2). To date, there is no evidence of eyelash pigment regrowth.

## Discussion

Tints are over-the-counter products whose primary ingredients are 3% hydrogen peroxide and p-phenylenediamine. They cause a semipermanent color change that only affects the cuticle layer of the hair.^4^ Accentuating eyelash color has been around since Cleopatra used makeup to highlight her periocular area. While there are no data to document the precise prevalence, eyelash tinting has become a technique routinely requested by patients to darken the lashes and obviate the need for mascara. There are few reported adverse reactions, and it remains a popular procedure readily offered by many estheticians in nonmedical facilities. There are no standardized protocols or governmental regulations, and in some states, it has been banned.^5^ Perhaps due to a profound inflammatory reaction,^6^ this is the first documented case of poliosis following semipermanent eyelash tinting. As poliosis can be permanent and can affect the patient’s quality of life, causing emotional distress and lowered self-esteem, we encourage other physicians to be aware of this potential occurrence. This patient declined any workup; however, if concerns exist and the patient has a history of sensitivity to cosmetic products or to permanent or semipermanent makeup that contains hydrogen peroxide or p-phenylenediamine, a patch test with the product should be considered.

## Conflicts of interest

None disclosed.
